# How to ask for and act on feedback: practical tips for medical students

**DOI:** 10.15694/mep.2018.0000063.1

**Published:** 2018-03-13

**Authors:** Kristen Davies, Jonathan Guckian

**Affiliations:** 1 Lancaster University; 2 Newcastle-Upon-Tyne Hospitals

**Keywords:** Feedback, Medical Students

## Abstract

This article was migrated. The article was marked as recommended.

Receiving feedback is an essential component of learning and can be invaluable to help you improve your knowledge and skills as you progress towards qualification as a doctor. Whilst formal feedback from assessments is commonplace, it can be daunting to approach others to provide you with feedback. Furthermore, it can be difficult to know how to act on the feedback once you have received it. In this article we look at how best for medical students to ask for feedback and act on the advice which is given to them with a unique point-of-view provided from a current clinical teaching fellow.

## Introduction

During your time at medical school you will receive feedback on your performance. This feedback can be invaluable to help you improve your knowledge and skills as you progress towards qualification as a doctor. This article offers tips on how to ask for feedback and how to act on the advice given to you.

## The purpose of feedback

Feedback is about providing you with information with the intention of closing the gap between actual and desired performance. Its purpose is to encourage you to reflect on your performance and how it can be improved (1). As a medical student you will be given regular feedback by supervisors at seminars and more formally as a part of formative assessments, with the aim of ‘closing the gap’ in preparation for summative assessments. Feedback on placement may be challenging to obtain due to the busy clinical environment. You may find it difficult to source the feedback you need or how to act on the feedback you receive.

## Asking for feedback

It can be a daunting task to approach a staff member to observe you and provide feedback on your performance. However, it is important to remember the bigger picture. Seeking feedback is a powerful initiative towards making improvements and knowing how to ask for feedback will make life easier for both you and your reviewer.

## Tips on asking for feedback (adapted from Algiraigri 2014 (2))

### 1) Assess which stage you are at

Before you can identify areas for improvement, you should perform a ‘self-assessment’ to understand your current levels of skills or knowledge. As medical professionals,might not be very good at this, however, as we tend to globally assess ourselves rather than looking at important areas in isolation.As a result, we may miss potential areas for improvement. One way to combat this is to break the scenario down into specific steps and analyse them individually. For example, when clerking a patient on the ward includes steps such as history taking, examination, clinical reasoning and verbally presenting the patient. By analysing each step individually, you are more likely to identify areas of weakness.

### 2) Set yourself goals

After you have identified the areas for improvement, set yourself distinctive goals with clear, attainable targets. Consider using a SMART framework when creating these:

The SMART Framework for creating goals from Doran GT (3):

• Specific
• Measureable
• Achieveable
• Relevant
• Time-bound

Distinctive goals can help motivate you in seeking feedback as you have a clear objective of what you want to achieve. Furthermore, research has suggested that medical students are able to improve performance by setting feedback-based goals (4).

### 3) Be specific

Share specific goals with your reviewer so they can pay particular attention to the areas you wish to improve. Not only will this help clarify what you wish to gain from asking for feedback, it will also help guide your reviewer in providing feedback. Several feedback models centre on aims-based discussion, such as the Agenda Led, Outcomes Based Assessment model (ALOBA) (5).

### 4) Clarify general feedback

Remember that you are part of the feedback process. Studies have suggested that medical students frame feedback as something which is done to them, rather than with them (6). Feedback should be a two-way process between you and your reviewer. You can ask your reviewer questions and discuss the feedback provided. Ask for clarification if needed, such as a demonstration of how an examination should have been done.

### 5) Involve your patients and peers

Whilst we may automatically assume that someone providing feedback should be a senior clinician, as they are more experienced, there is value in gaining feedback from a variety of sources. Your medical student colleagues may have a better idea of the stage you are at and what you have been taught and can assess you against that standard (7). Similarly, your patients can provide feedback about your communication skills and how you made them feel, which is a unique insight. The benefit of having a number of people observing you means that you can delegate areas for review to each person, so everyone has a clear objective of what they are doing.

## Asking for feedback: A Teaching Fellow’s View

### Avoid feedback for feedback’s sake

This may appear to be surprising advice from a teaching fellow, but faculty do not always provide the best sources of feedback. Pre-arranged clinical observations by faculty often create an artificial safe-zone within the ward environment, making performance unrealistic and therefore influencing feedback. Instead, look to the clinicians on your ward. Build relationships with them and become part of the ward team. If feedback is interwoven with clinical activities, it becomes more of a natural process rather than a distractor (8).

### Remember that feedback is a two-way street

The idea that feedback is a unilateral process dictated to the learner should be challenged. Such an approach may risk misinterpretation of key messages, and is less likely to lead to change (9). Feedback should instead be a collaborative undertaking. This may take extra planning and possibly courage, however provides more context to the feedback, leads to deeper understanding and offers further opportunity for facilitated reflection (10) Try speaking to the colleague feeding back to you in advance, and indicate your preference for a more natural conversation than a ‘lesson’. Educators may well prefer this approach.

## Acting on feedback

Now that you have received your feedback, it is important that you reflect and change your behaviour in some way. This is not only an important step with regard to improvement, but it is also highlighted by the GMC as a mandatory skill in order to demonstrate that you are fit to practice (11).

## Tips on acting on feedback

### 1. Responding to feedback

The process of receiving feedback can be challenging as you are being reviewed, either positively or negatively, and potentially in front of an audience. Try to view receiving feedback as an opportunity for personal improvement and remember that everyone has room for improvement, regardless of their position. Try not to take the comments personally, as they are a reflection of actions requiring improvement rather than of yourself or your character.

### 2. Note the key points

Make sure you write down a summary of the feedback you receive so you can effectively reflect on the experience. It’s important to realise that not everyone is trained in giving feedback effectively, so discuss with your reviewer the key points to take away.

### 3. Reflect on the experience

Once you have your feedback summary, incorporate it into your reflection on the experience. Reflection is the most important step in learning from clinical experiences. Ask yourself questions about the experience, what do you think went well and what could you improve? Some examples of questions to ask yourself during your reflection can be found belwo. See if your own self-evaluation matches with the feedback you received. We may not be aware of our mistakes until they are pointed out by others.

### Example questions for reflection (adapted from Kolb’s learning cyctitlee (12))

1. Description of the experience: What happened? Where did it take place?
2. Thinking about the experience: What were your thoughts and feelings before, during and after the experience? How did the experience make you feel?
3. Drawing conclusions: What were you trying to achieve? What knowledge did you gain? What skills did you acquire? Where could you improve?
4. Moving forward: How can this learning experience benefit you in the future?

### 4. Adjust your goals

Now that you have received and reflected on your feedback, go back to your original goals and assess whether you have achieved them. Reevaluate the goals you have set and identify new areas for improvement. Remember that successful doctors do not stop when they have received positive feedback, instead they continue to strive for constant improvement.

## Acting on feedback: A Teaching Fellow’s View

### Be aware of your avoidance behaviours

All learners could be described to have a ‘psychological immune system’ (13), which can manifest in an avoidance response to negative feedback. Feedback is an inherently emotional process, sometimes not unlike breaking bad news to a patient. Being aware of areas that you tend to neglect, or responses you may take personally, is the first step in addressing challenging feedback. Ensure feedback is written down, and take as much time as you need to depersonalise the feedback if necessary.

### Reflect on the ‘good stuff’

Some learners are inherently self-critical. This idea is often seen in action with the use of Pendleton’s model of feedback (5), as learners frequently ignore what they did well and move directly to areas for improvement. Whilst the point of feedback is indeed reflection leading to change, this does not always need to be negative. Learning from the actions we have done well can be just as valuable as from our mistakes. Taking appropriate time in your portfolio or learning diary to write down one ‘good thing’ or improvement every day may sound immature, however it is the sign of a reflective practitioner building on positive foundations.

## Conclusion

Feedback is a continuous, collaborative process which is the cornerstone of learning from both positive and negative experiences. By setting yourself goals, taking the initiative and asking for feedback on your performance, you can take control of your learning. Reflecting on this feedback will facilitate consistent improvement in performance and allow you to practice a skill which is so vital in a career in medicine.

## Take Home Messages

Before asking for feedback, assess which stage you are at and approach a variety of people (peers, patients) with specific feedback objectives so both you and your assessor are clear about what you are doing.

When acting on feedback, note down the key points given to you and reflect on your experience and adjust your personal goals accordingly.

## Notes On Contributors

Kristen Davies is a final-year medical student at Lancaster University. He is the co-founder of the Lancaster University Peer-Assisted Learning Society (LUPALS) and has previously been awarded the BMJ Clegg Scholarship in Medical Education.

Dr Jonathan Guckian is a Clinical Teaching Fellow at Newcastle Upon Tyne Hospitals Trust. He is also a Director of the Association for the Study of Medical Education (ASME) and founder of Medisense, a prominent online medical education platform
