# Supplementary material for: How to ask for and act on feedback: practical tips for medical students
Source: MedEdPublish (2016). 2018 Mar 13;7:63. [Version 1] doi: 10.15694/mep.2018.0000063.1 (PMC10711946; doi:10.15694/mep.2018.0000063.1)
Supplement: Peer review history

# Reviewer report: Reviewer response for version 1

**DOI:** 10.21956/mep.19532.r28917

**Published:** 2018-04-20

**Authors:** Maurizio Vertemati

**Affiliations:** 1 Dipartimento di Scienze Biomediche e Cliniche "L. Sacco"

This review has been migrated. The reviewer awarded 3 stars out of 5 An interesting manuscripts that underlines how students (and also teachers) can learn more quickly or perform at a higher level if appropriate feedback is given. Feedback is constantly a work in progress. All sections are very interesting. This article should be recommended in academic setting.

Reviewer Expertise:

NA

No decision status is available

# Reviewer report: Reviewer response for version 1

**DOI:** 10.21956/mep.19532.r28915

**Published:** 2018-03-29

**Authors:** Susmita Reddy Karri

**Affiliations:** 1 Topiwala national medical college and BYL nair charitable hospital, mumbai. india

This review has been migrated. The reviewer awarded 4 stars out of 5 A good article which emphasises that feedback is a two-way communication. I really liked the "Fellow teacher's" point of view. A must read for all students.

Reviewer Expertise:

NA

No decision status is available

# Reviewer report: Reviewer response for version 1

**DOI:** 10.21956/mep.19532.r28916

**Published:** 2018-03-23

**Authors:** Konstantinos Fragkos

**Affiliations:** 1 University College London

This review has been migrated. The reviewer awarded 4 stars out of 5 This article has practical tips that are absolutely essential. Feedback is necessary for continuing professional development. Asking for it and using it effectively can be more tricky than thought. I think this papers adds to this topic.

Reviewer Expertise:

NA

No decision status is available

# Reviewer report: Reviewer response for version 1

**DOI:** 10.21956/mep.19532.r28914

**Published:** 2018-03-14

**Authors:** J.M.Monica van de Ridder

**Affiliations:** 1 Spectrum Health - Michigan State University College of Human Medicine

This review has been migrated. The reviewer awarded 3 stars out of 5 It is nice to have a tips and guidelines contribution on seeking feedback and acting on feedback. The authors review the purpose of feedback, explain why it is important to ask for feedback, and give practical tips derived form an article. In the second part of the topic is approached from a teaching fellow’s perspective.The author’s state that asking for feedback can be very powerful. I think it is important to take into consideration that this is dependent on the culture of the setting in which feedback is sought. In some professional cultures seeking feedback is viewed as a weakness. It is perceived that you are insecure, and that you seek feedback to receive a confirmation. In other professional cultures feedback seeking is seen as a strength. So for a learner it might be important to explore the culture of the learning environment, before seeking feedback. Otherwise the feedback seeking might work against you instead of facilitating your learning.(1). Often when supervisors, chairs, people higher in the hierarchy seek feedback this is a sign that feedback seeking is welcomed and viewed as a strength.In the acting on feedback from the teaching fellow’s perspective it is advised that it is important to ‘take as much time as you need to depersonalise the feedback if necessary’. This notion is very important, because when the learner does not take the feedback as a personal ‘attack’ but recognizes it given with the intent to improve behavior, the likelihood that the feedback will be used is higher. I think it will be valuable for students and other readers to have specific tips they can use on how to depersonalize the received feedback.It is nice that specific guidelines for students are given on how to ask and act upon feedback. I accentuates that feedback is a shared responsibility. The guideline are helpful for both feedback givers and seekers. I will encourage the students who are taking an intersession on feedback seeking to read the guidelines. 1. Ashford SJ, Blatt R, VandeWalle D. Reflections on the looking glass: A review of research on feedback-seeking behavior in organizations. Journal of Management. 2003;29(6):773-99.

Reviewer Expertise:

NA

No decision status is available

# Reviewer report: Reviewer response for version 1

**DOI:** 10.21956/mep.19532.r28918

**Published:** 2018-03-14

**Authors:** BALAJI ARUMUGAM

**Affiliations:** 1 TAGORE MEDICAL COLLEGE AND HOSPITAL

This review has been migrated. The reviewer awarded 4 stars out of 5 The section which I liked the most is "Acting on feedback". Feedback in medical education is an integral and important element of teaching as it encourages and enhances the learners' knowledge, skills and professional performance. In addition to it, it increases the improvement of the performance of the learners with the basic aim of helping them achieve their goals in addition to the educational objectives. The potential of feedback can be maximized provided the teacher is receptive to suggestions for change and willing to improve.

Reviewer Expertise:

NA

No decision status is available
